# Supplementary material for: Long-term musical training can protect against age-related upregulation of neural activity in speech-in-noise perception
Source: PLoS Biol. 2025 Jul 15;23(7):e3003247. doi: 10.1371/journal.pbio.3003247 (PMC12262870; doi:10.1371/journal.pbio.3003247)
Supplement: S1 Text — (DOCX) [file pbio.3003247.s001.docx]

**Text S1**

**Effect of years of education on the behavioral performance and neural responses in the audiovisual speech-in-noise perception task**

We found that years of education including both formal and informal education were significantly different between ONM and OM. Due to historical reasons in China, all recruited older adults discontinued formal education at the same time (1960s-1970s). Importantly, our interviews revealed fundamental qualitative differences in educational backgrounds between older groups. Most older musicians attended specialized institutions, such as music middle schools, music high schools, or conservatories, with curricula focused on music training rather than on the broader academic subjects (e.g., language and science) typical of standard education. This qualitative difference means that years of education cannot be directly compared between musicians and non-musicians, even when the nominal duration is similar. Consequently, self-reported years of education do not reliably represent educational attainment in our older cohort.

Furthermore, years of education were highly correlated with the group variable between OM and ONM (point-biserial correlation r = 0.64), and were correlated with the group variable between older subjects and young participants (ONM vs. YNM: $r_{pb}$= 1; OM vs. YNM: $r_{pb}$= 0.74). Therefore, it is not suitable to put the years of education variable into the analyses as a control variable because of the collinearity problem.

Nonetheless, to explicitly address the potential effect of years of education on our results, we performed supplementary analysis to investigate the effect of years of education on the behavioral performance, BOLD activation, task-induced functional connectivity, and resting-state functional connectivity. We included the years of education in the mixed-designed ANOVA. We found years of education was not significant (behavioral: all p > 0.522; task-induced functional connectivity: all $P_{fdr}$ > 0.209; resting-state functional connectivity: all $P_{fdr}$ > 0.649). Therefore, years of education did not influence the behavioral performance and neural responses in this study.
