# Supplementary figures and images for: Long-term musical training can protect against age-related upregulation of neural activity in speech-in-noise perception
Source: PLoS Biol. 2025 Jul 15;23(7):e3003247. doi: 10.1371/journal.pbio.3003247 (PMC12262870; doi:10.1371/journal.pbio.3003247)

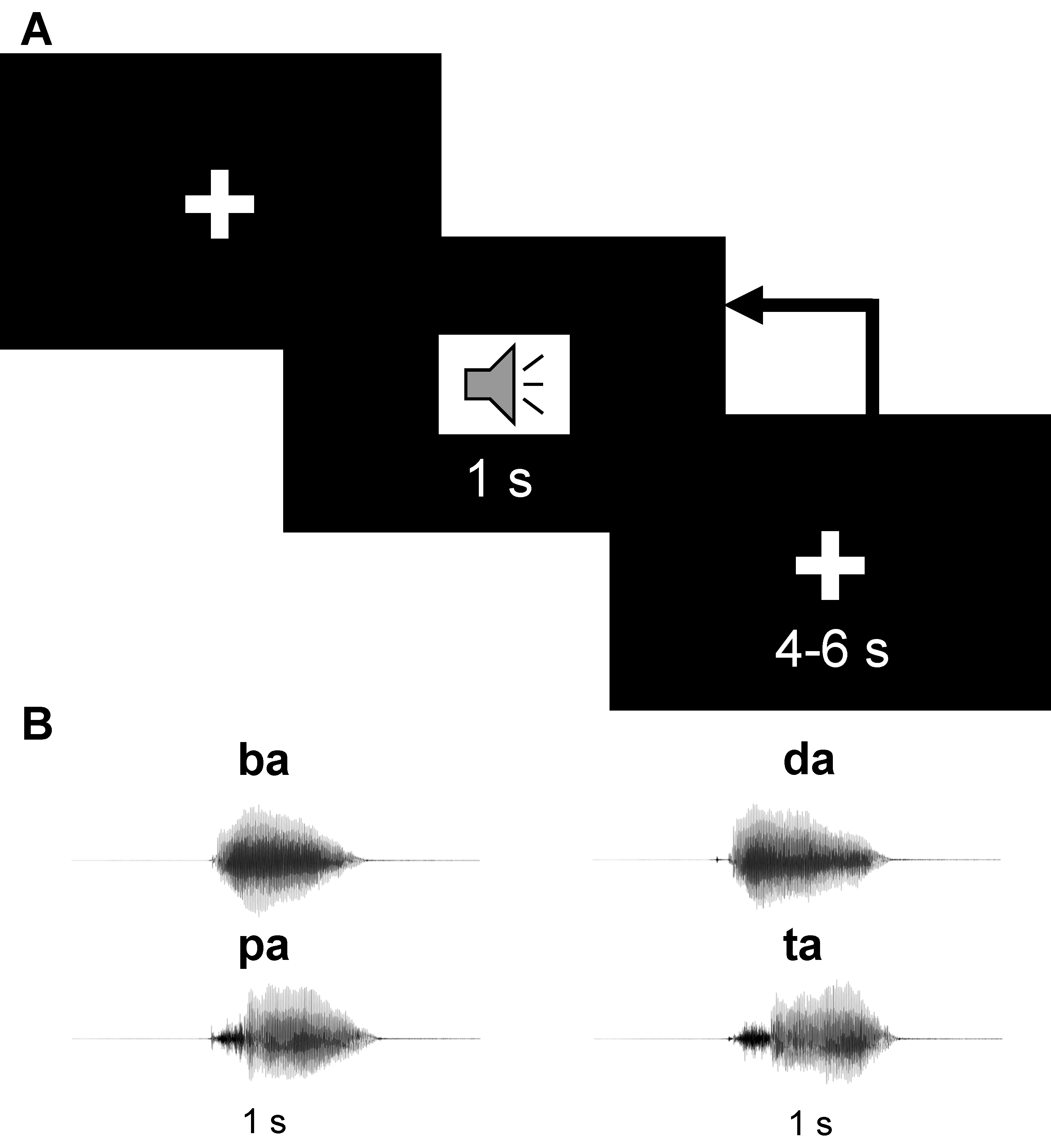

Supplement: S1 Fig — (TIF) [file pbio.3003247.s002.tif]

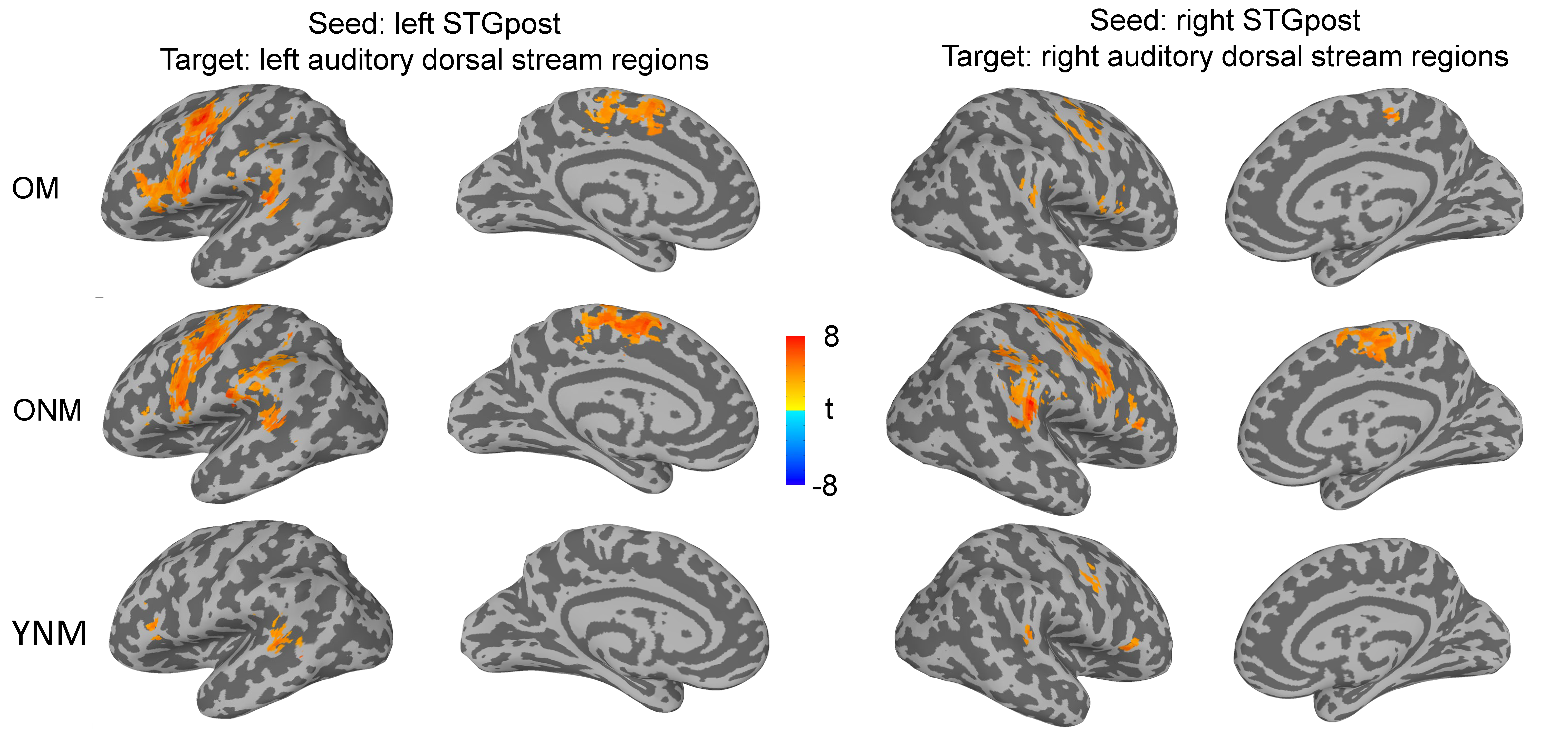

Supplement: S2 Fig — (TIF) [file pbio.3003247.s003.tif]

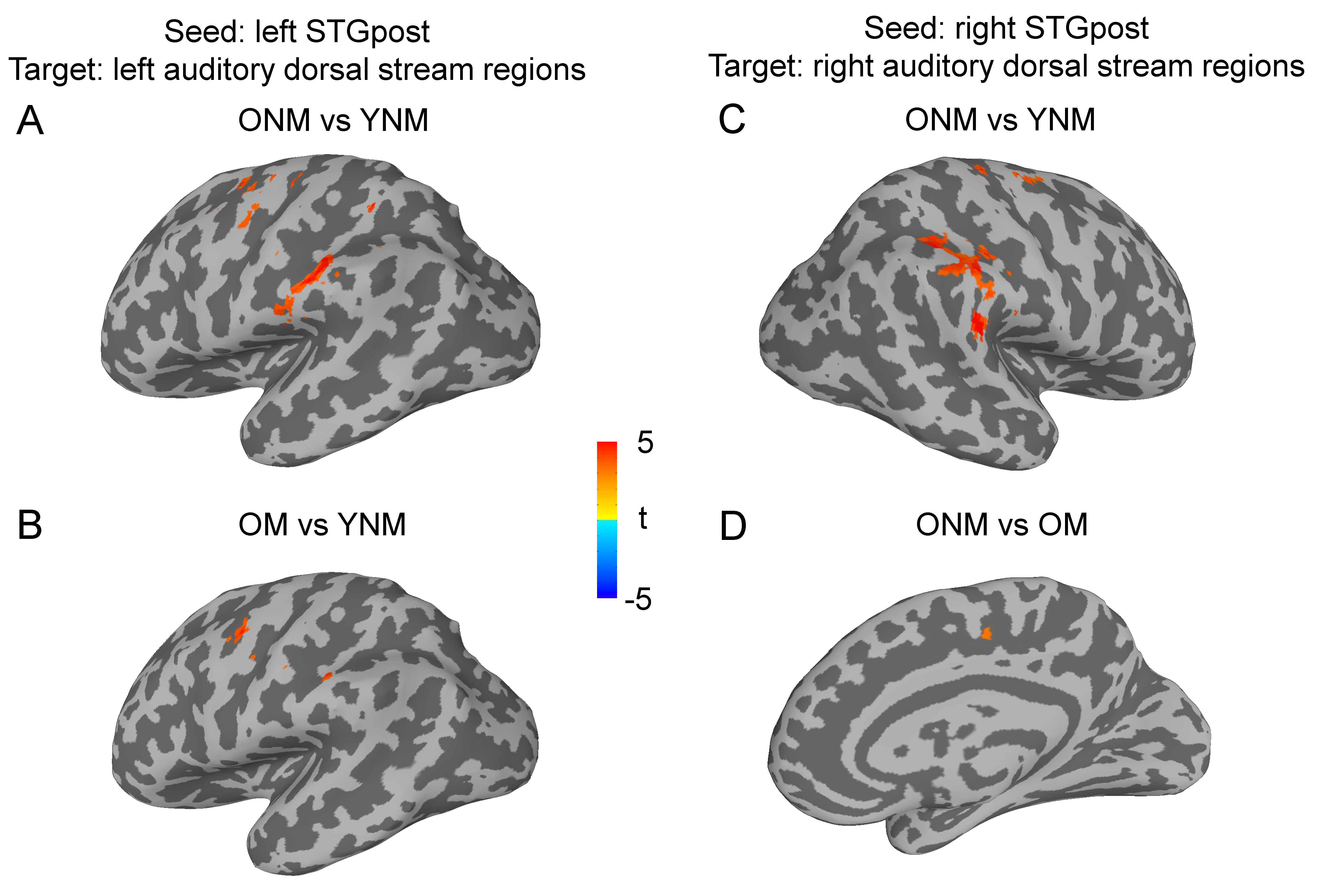

Supplement: S3 Fig — Older non-musicians showed greater functional connectivity in left and right auditory dorsal stream regions than young non-musicians (A, C), older musicians (D). Older musicians showed greater functional connectivity in left auditory dorsal stream regions than young non-musicians (B). Pfwe < 0.05; STGpost, posterior superior temporal gyrus; OMs, older musicians; ONMs, older non-musicians; YNMs, young non-musicians. (TIF) [file pbio.3003247.s004.tif]

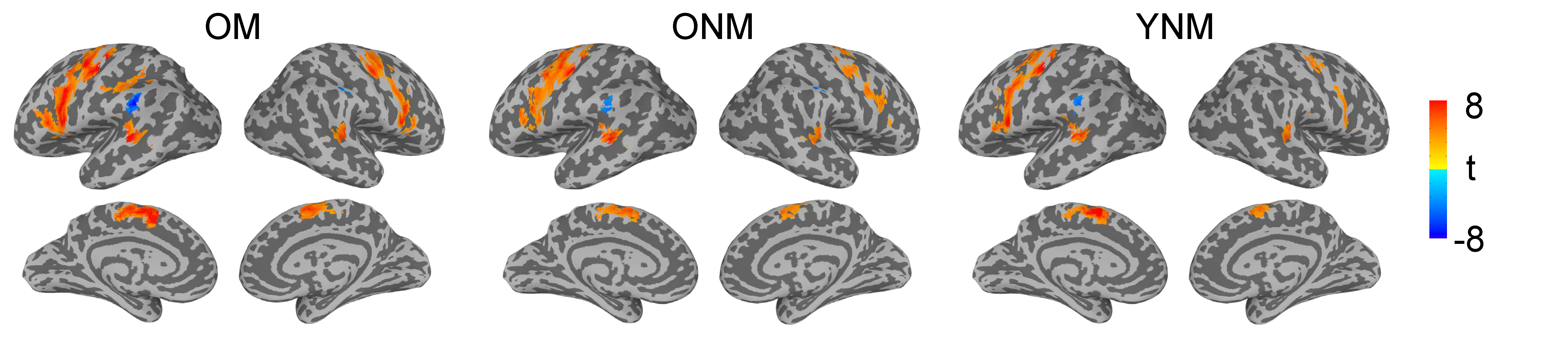

Supplement: S4 Fig — (TIF) [file pbio.3003247.s005.tif]
