# Supplementary material for: Long-term musical training can protect against age-related upregulation of neural activity in speech-in-noise perception
Source: PLoS Biol. 2025 Jul 15;23(7):e3003247. doi: 10.1371/journal.pbio.3003247 (PMC12262870; doi:10.1371/journal.pbio.3003247)
Supplement: S1 Table — The F values and associated P values represent the main effect of the group from a mixed-design ANOVA. The reported P values have been corrected for multiple comparisons across the ROIs using the FDR method. The t values and associated P values are from post hoc pairwise comparisons with FDR correction. (DOCX) [file pbio.3003247.s006.docx]

**Table S1**. Mixed-design ANOVA and post hoc analysis of task-induced functional connectivity. The F values and associated P values represent the main effect of the group from a Mixed-design ANOVA. The reported P values have been corrected for multiple comparisons across the ROIs using the FDR method. The t values and associated P values are from post hoc pairwise comparisons with FDR correction.

| Seed: LSTG | Target regions | Group main effect  $F_{2,69}$($P_{fdr}$) | OMs vs. ONMs  $t_{69}$($P_{fdr}$) | OMs vs. YNMs  $t_{69}$($P_{fdr}$) | ONMs vs. YNMs  $t_{69}$($P_{fdr}$) |
| --- | --- | --- | --- | --- | --- |
|  | L SMA | 16.00(<0.001) | 2.05(0.044) | -3.54(0.001) | -5.59(<0.001) |
|  | L SMG | 12.64(<0.001) | 2.38(0.020) | -2.64(0.015) | -5.03(<0.001) |
|  | L PrCGsup | 25.02(<0.001) | 2.46(0.016) | -4.51(<0.001) | -6.97(<0.001) |
|  | L SM | 13.55(<0.001) | 0.63(0.533) | -4.16(<0.001) | -4.79(<0.001) |
|  |  |  |  |  |  |
| Seed: RSTG | Target regions |  |  |  |  |
|  | R SMA | 7.10(0.002) | 2.24(0.042) | -1.50(0.137) | -3.74(0.001) |
|  | R SMG | 18.83(<0.001) | 2.68(0.009) | -3.44(0.001) | -6.12(<0.001) |
|  | R PrCGsup | 15.39(<0.001) | 3.13(0.004) | -2.40(0.019) | -5.53(<0.001) |
|  | R SM | 3.69(0.030) | 1.85(0.104) | -0.80(0.425) | -2.65(0.030) |
