# Supplementary material for: Long-term musical training can protect against age-related upregulation of neural activity in speech-in-noise perception
Source: PLoS Biol. 2025 Jul 15;23(7):e3003247. doi: 10.1371/journal.pbio.3003247 (PMC12262870; doi:10.1371/journal.pbio.3003247)
Supplement: S2 Table — The F values and associated P values represent the main effect of the group from a mixed-design ANOVA. The reported p values have been corrected for multiple comparisons across the ROIs using the FDR method. The t values and associated P values are from post hoc pairwise comparisons with FDR correction. (DOCX) [file pbio.3003247.s007.docx]

**Table S2**. One-way ANOVA and post hoc analysis of intrinsic functional connectivity. The F values and associated P values represent the main effect of the group from a Mixed-design ANOVA. The reported p values have been corrected for multiple comparisons across the ROIs using the FDR method. The t values and associated P values are from post hoc pairwise comparisons with FDR correction.

| Seed: **LSTG** | Target regions | Group main effect  $F_{2,69}$($P_{fdr}$) | OMs vs. ONMs  $t_{69}$($P_{fdr}$) | OMs vs. YNMs  $t_{69}$($P_{fdr}$) | ONMs vs. YNMs  $t_{69}$($P_{fdr}$) |
| --- | --- | --- | --- | --- | --- |
|  | L SMA | 6.20(0.010) | -0.66(0.511) | -3.52(0.002) | -2.11(0.058) |
|  | L SMG | 5.68(0.010) | -0.17(0.869) | -3.36(0.004) | -2.38(0.031) |
|  | L PrCGsup | 4.37(0.022) | -0.43(0.668) | -2.96(0.013) | -1.87(0.099) |
|  | L SM | 2.80(0.068) | -0.55(0.585) | -2.35(0.064) | -1.33(0.281) |
|  |  |  |  |  |  |
| Seed: **RSTG** | Target regions |  |  |  |  |
|  | R SMA | 5.23(0.015) | 0.05(0.964) | -3.20(0.006) | -2.42(0.027) |
|  | R SMG | 5.27(0.015) | -0.81(0.420) | -3.22(0.006) | -1.78(0.120) |
|  | R PrCGsup | 3.31(0.050) | 0.06(0.954) | -2.54(0.040) | -1.94(0.085) |
|  | R SM | 3.12(0.050) | -0.71(0.481) | -2.47(0.048) | -1.29(0.301) |
