# Supplementary material for: Long-term musical training can protect against age-related upregulation of neural activity in speech-in-noise perception
Source: PLoS Biol. 2025 Jul 15;23(7):e3003247. doi: 10.1371/journal.pbio.3003247 (PMC12262870; doi:10.1371/journal.pbio.3003247)
Supplement: S3 Table — The F values and associated P values represent the main effect of the group from a mixed-design ANOVA. The reported P values have been corrected for multiple comparisons across the ROIs using the FDR method. The t values and associated P values are from post hoc pairwise comparisons with FDR correction. (DOCX) [file pbio.3003247.s008.docx]

**Table S3**. Mixed-design ANOVA and post hoc analysis of BOLD activation. The F values and associated P values represent the main effect of the group from a Mixed-design ANOVA. The reported P values have been corrected for multiple comparisons across the ROIs using the FDR method. The t values and associated P values are from post hoc pairwise comparisons with FDR correction.

|  | Group main effect  $F_{2,69}$($P_{fdr}$) | OMs vs. ONMs  $t_{69}$($P_{fdr}$) | OMs vs. YNMs  $t_{69}$($P_{fdr}$) | ONMs vs. YNMs  $t_{69}$($P_{fdr}$) |
| --- | --- | --- | --- | --- |
| L SMA | 2.29(0.241) | -2.05(0.132) | -0.51(0.613) | 1.54(0.190) |
| L SMG | 1.75(0.241) | -1.87(0.199) | -0.80(0.426) | 1.07(0.426) |
| L PrCGsup | 0.24(0.791) | -0.59(0.832) | -0.59(0.832) | -0.00(0.999) |
| L SM | 2.57(0.241) | -2.27(0.080) | -1.12(0.265) | 1.14(0.265) |
| R SMA | 1.19(0.356) | -1.43(0.343) | -1.21(0.343) | 0.22(0.830) |
| R SMG | 1.93(0.241) | -1.96(0.163) | -1.10(0.395) | 0.86(0.395) |
| R PrCGsup | 2.29(0.241) | -0.98(0.332) | -2.14(0.108) | -1.16(0.332) |
| R SM | 1.87(0.241) | -1.53(0.194) | -1.78(0.194) | -0.25(0.803) |
