# Supplementary material for: Long-term musical training can protect against age-related upregulation of neural activity in speech-in-noise perception
Source: PLoS Biol. 2025 Jul 15;23(7):e3003247. doi: 10.1371/journal.pbio.3003247 (PMC12262870; doi:10.1371/journal.pbio.3003247)
Supplement: Attachment — Submitted filename: responses to reviewers_20250420.docx [file pbio.3003247.s011.docx]

We thank the editor and two reviewers for their constructive feedback. In response to their thoughtful suggestions, we have revised the manuscript. Below we provide point-by-point responses to the concerns and questions raised. We believe these revisions have significantly improved the manuscript, and we hope all issues have been addressed satisfactorily. Thank you for the opportunity to resubmit our work.

REVIEWS:

Reviewer #1: This article by Zhang and colleagues reports an fMRI study that compares brain responses during syllable identification under noise conditions across three groups of participants: Older musicians (OMs), older non-musicians (ONMs) and young non-musicians (YNM). The research builds upon the broader context of investigating  how cognitive resources derived from positive lifestyle factors, such as music learning,  influence compensatory neural mechanisms associated with aging and observed in numerous previous studies.
The authors compare two possibilities: the "Bolster Compensation Hypothesis" suggesting that the greater availability of cognitive resources increase compensatory activity in older adults during cognitive tasks; and the "Hold-Back Upregulation hypothesis" suggesting instead that high cognitive resources mitigate age-related decline in perception and cognition, thus decreasing compensatory activity compared to age-matched control participants.
The authors consider a task of syllable identification (four choice) in noise, with three noise levels (-8db, 0 db, 8 db). As a measure of compensatory neural activity, the authors use the psychophysiological interaction between speech-related regions (posterior STG) and a set of parietal and frontal regions belonging to the dorsal auditory stream. Increased PPI is taken as a measure of increased compensatory activity, and thus an increase across groups (YNM < ONM < OM) is taken to support the first hypothesis, while intermediate levels for OMs (OM - YNM < ONM - YNM) are taken as evidence that older participants with greater cognitive resources are more similar than age-matched controls to young participants and thus support the second hypothesis. Reported behavioural and neural results support this latter hypothesis, highlighting the role of compensatory neural mechanisms in managing cognitive challenges associated with aging.

The study provides novel evidence suggesting that music training may play a significant role in improving neurocognitive resilience and it may be relevant for researchers studying the relationship between musicality and age-related cognitive changes. In general, the study is well-designed; however, several aspects require clarifications and improvements. These aspects concern the study description, which can be improved easily, but also relevant methodological choices, which now seem ad hoc and require further justification. I detail my considerations below:

**Response:** Thank you for a great summary and positive comments. Here are point-to-point responses to your suggestions.

- Title (and scope): I would prefer a title that is closer to the experimental manipulation and experiments conducted, and thus I would replace "Cognitive reserves" with "Musical training" or "Musical expertise" as the equivalence Musical training = Higher cognitive reserves is an assumption that has not been tested in this study.

**Response:** We appreciate the reviewer’s insightful suggestion regarding the manuscript title. We agreed that we did not directly test higher cognitive reserves in older musicians. We have changed the title to “Musical expertise holds back age-related upregulation of neural activities in speech-in-noise perception”.

- The experiment, task and rationale for the analyses need to be introduced more extensively before the results description. Currently, it is not possible to follow the results section without first reading the methods section.

**Response:** Thanks for the suggestion. We have expanded the introduction in each result section.

Behavioral results:

Line 131-134: The two-way mixed-design analyses of variance (ANOVA) with group (OM/ONM/YNM) and SNR (8, 0, -8 dB) as between- and within-subject factor, respectively, revealed main effects of group and SNR, and an interaction between group and SNR.

TiFC results:

Line 156-159: We performed gPPI analysis to investigate how aging and musical training modulated the TiFC within the bilateral auditory dorsal streams, which are crucial in speech perception [36,37,39]. The gPPI analysis quantifies whether the task demands modulate functional connectivity between two brain regions [40].

Line 182-185: Because standard parametric tests (e.g., ANOVA or linear mixed models) do not directly assess whether the magnitude of the difference between ONMs and YNMs exceeds that between OMs and YNMs, we employed a nonparametric bootstrap approach with 10,000 iterations (for details, see methods).

Spatial alignment results:

Line 229-233: Next, we extended our analysis to quantify spatial shifts in connectivity peaks within the left PrCGsup. While ISPC measures overall spatial neural alignment between older and young adults, it does not capture specific reorganization patterns. By extracting the mean coordinates of the top 10% of voxels with the highest TiFC, we precisely mapped connectivity spatial shifts in older adults relative to young non-musicians (Fig. 4D).

RSFC results:

Line 262-263: RSFC was measured by calculating Pearson correlation coefficients between voxel-wise time series in the same seed and target regions as used in the gPPI analysis.

- Pg. 3, 77-81:  Fig.1D should be Fig. 1C,  Fig 1E should be Fig. 1D

**Response:** Thanks for pointing out. We have corrected them.

- Pg. 3 91: SIN perception: "SIN" should be defined

**Response:** Thanks for pointing out this. We have added the full definition of "SIN" (speech-in-noise).

Line 84-86: In comparison, older adults tend to over-recruit dorsal stream brain regions to compensate for deficits in speech-in-noise (SIN) perception (Fig. 1A) [32–34].

- It is unclear if the behavioural data (and their analysis) refer to data collected during the fMRI measurements or outside the scanner.

**Response:** The behavioral data are data collected during the fMRI measurements. We have added this specification in both the Methods and Results sections:

Line 129-131: First, we examined whether long-term musical training protected against age-related declines in SIN perception among older adults by comparing the accuracy of OM, ONM and YNM groups in identifying syllables amidst background noise during fMRI acquisition.

Line 461-462: Mixed-design ANOVAs were performed to investigate the main effect of group on behavioral performance collected during fMRI scanning.

- The SNR manipulation is interesting and the beahvioural results provide a perfect context for the analysis of the fMRI data. They show that OM performed worse than YNMs under all SNRs (as expected) but better than ONMs only under the intermediate and high SNR. I wonder why this SNR dependence has not been explored more extensively in the analysis of fMRI data as well.

**Response:** In the revised manuscript, we explored this SNR dependence. We performed two-way mixed ANOVAs, which include both group and SNR factors for all neural measures. Main effect of SNR is significant on BOLD activation ($P_{fdr}$ < 0.047 for all regions except left superior precentral gyrus. $P_{fdr}$ = 0.171), but no interaction effect was found (all $P_{fdr}$ > 0.981). For functional connectivity, main effect of SNR was not significant (all $P_{fdr}$ > 0.708), while the interaction effect was significant in left brain (all $P_{fdr}$ < 0.05). However, since our hypotheses focus on group differences, the interaction effect are not further analyzed.

We add the SNR related results in the results part:

Line 164-166: Main effect of SNR was not significant (all $P_{fdr}$ > 0.708), while the interaction effect was significant in left hemisphere (all $P_{fdr}$ < 0.05). Since our hypotheses focus on group differences, the interaction effect is not further analyzed.

Line 281-283: However, we did not observe any BOLD activation difference between the three groups (for statistics, see Supplementary Table 3) or significant interaction effect between SNR and groups (all $P_{fdr}$ > 0.981).

Finally, we agree that the interaction of task difficulty and cognitive reserve is an important avenue for future work. We have noted this in the Discussion:

Line 406-407: Future studies should explore how varying task difficulty (e.g., different SNRs) interacts with cognitive reserve to shape age‑related changes in neural activity.

- The main analysis of the fMRI data relies on ROI-based PPI. However, PPI (and connectivity analyses in general) may be difficult to interpret without contextualizing the effects with a conventional voxel-wise analysis of brain activation and brain activation differences.

**Response:** Thank you for the suggestion. We conducted voxel-wise analyses of BOLD activation within the auditory dorsal stream ROIs and present the activation maps for all three groups in Supplementary Figure S4 ($P_{fwe}$ < 0.05). Consistent with ROI analysis, no group difference was found in BOLD activation. We added the description in the results section.

Line 283-285: Voxel-wise analysis revealed significant activation in auditory dorsal stream regions from all three groups (Fig. S2), while the group difference in regional activation was not significant.

Fig.S4. Voxel-wise BOLD activation in auditory dorsal stream for three groups. $P_{fwe}$ < 0.05; OMs, older musicians; ONMs, older non-musicians; YNMs, young non-musicians

Line 36-44 in supplementary materials for method description:

**Group analysis of voxel-wise BOLD and functional connectivity**

We used ANOVA from AFNI program 3dMVM to examine group difference of voxel-wise BOLD activation and functional connectivity as measured with gPPI in auditory dorsal stream among the three groups (ONM, YNM, and OM). Model were constructed with the within-subject factor (SNRs) and between-subject group factors (ONM, YNM, and OM). Group activation and functional connectivity were tested using general linear model. Multiple comparisons were corrected using 3dClustSim (“fixed” version) with real smoothness of data estimated by 3dFWHMx (acf method) [1]. A total of 10,000 Monte Carlo simulations were performed to get the cluster threshold (α = 0.05 family-wise error corrected). Results were visualized onto an inflated cortical surface using surface mapping with AFNI (SUMA).

- The authors only report regional activations in a suppl. Table. However, I would prefer to see the activation maps for the three groups and be able to judge whether these maps, at least t to inspect whether the activation across the three groups is qualitatively similar (that is are all ROIs active in the three groups?).
Furthermore, I think that a GLM-based analysis of the voxel-wise between-group differences and the interaction between group and SNR could be very informative and should be presented to the readers.

**Response:** We thank the reviewer for the suggestion. We have added Supplementary Figures S2-S4, which present the voxel-wise functional connectivity map for each group (Fig. S2), between-group differences of functional connectivity map (Fig. S3), and activation map for each group (Fig. S4). Consistent with our ROI-based analysis, we did not find clusters showed significant group difference of BOLD activation. And we did not find significant interaction effect on BOLD activation and functional connectivity. We mentioned these voxel-wise map in the results section.

Line 168-173: We found a main effect of group on TiFC within the bilateral auditory dorsal streams (LpSTG-LSMA, LSMG, LPrCGsup, LSM; RpSTG-RSMA, RSMG, RPrCGsup, RSM, for voxel-wise functional connectivity map of the three groups, see **Fig. S2**). For connectivity that showed a significant group effect after FDR correction, pairwise comparisons revealed stronger TiFC in ONMs than young adults (LpSTG-LSMA, LSMG, LPrCGsup, LSM; RpSTG-RSMA, RSMG, RPrCGsup, RSM, **Fig. 3C**, **Supplementary Table 1**, for voxel-wise analysis, see **Fig. S3A, C**).

Line 179-181: Both OMs and ONMs showed stronger connectivity than YNMs in the left hemisphere (LpSTG-LSMA, LSMG, LPrCGsup, LSM, **Fig. 3C**, for statistics, see **Supplementary Table 1,** for voxel-wise analysis, see **Fig. S3A, B, C**).

Line 188-190: The ONMs showed stronger connectivity in the right hemisphere than OMs (RpSTG-RSMA, RSMG, RPrCGsup**,** for voxel-wise analysis, see **Fig. S3D**) and YNMs (RpSTG-LSMA, RPrCGsup, RSMG, RSM).

Fig.S2. Voxel-wise functional connectivity as measured with gPPI in auditory dorsal stream seeded from left and right posterior STG for three groups. $P_{fwe}$ < 0.05; STGpost, posterior superior temporal gyrus; OMs, older musicians; ONMs, older non-musicians; YNMs, young non-musicians

Fig.S3. Voxel-wise group comparison of functional connectivity. Older non-musicians showed greater functional connectivity in left and right auditory dorsal stream regions than young non-musicians (A, C), older musicians (D). Older musicians showed greater functional connectivity in left auditory dorsal stream regions than young non-musicians (B). $P_{fwe}$ < 0.05; STGpost, posterior superior temporal gyrus; OMs, older musicians; ONMs, older non-musicians; YNMs, young non-musicians.

-Related to the GLM analysis, is was unclear what are the "Six conditions of four syllables" (pg. 14, line 420). More in general, the experimental design, the timing of the stimuli and the separation of condition in blocks was not clear to me, and it could be improved (maybe with an illustration?).
- Pg 15, 450: Unclear: "Mixed-design ANOVAs (group*SNRs) were performed for A and visual enhancement"

**Response:** Thank you for catching these unclear points. We apologize for these miswritten parts. We’ve corrected the GLM analysis description to clarify that we modeled "four syllables under three SNRs along with six motion parameter regressors." For the ANOVA analysis, we’ve investigated "group and SNR effects on neural activation and functional connectivity."

Line 478-479: Four syllables under three SNRs and six regressors corresponding to motion parameters were entered into the analysis.

Line 461-462: Mixed-design ANOVAs were performed to investigate the main effect of group on behavioral performance collected during fMRI scanning.

We have added an illustration for trials and syllables in the supplementary materials (Fig.S1). Also, we have improved the description of the experiment design.

Line 456-459: Each participant completed four blocks. Each block contained 60 stimuli trials (3 SNRs × 4 syllables × 5 repetitions). As shown in Fig.S1A, each trial presented a 400 ms syllable (see waveform examples in Fig. S1B) followed by a jittered intertrial interval of 4–6 s (0.5 s steps). Four syllables under 3 SNRs were pseudo-randomly ordered within blocks.

- For the PPI analysis, it is stated "PPI regressors, the seed time series, and the regressors of the original GLM model were included in the model". What did enter the ANOVA analysis? Are the betas for the PPI regressors? Were they significant? Or, in other words, did the PPI regressor explain additional variance that was not explain by the original GLM predictors?

**Response:** Thanks for the question. Yes, betas for the PPI regressors were included in the analysis. Here, we provided the voxel-wise map (Please see the Fig S2 above). From the map, PPI regressors were significant in all ROIs in older adults. We have also clarified that in the Methods section.

Line 503-505: To specifically investigate how aging and musical training modulate regional activities in the auditory dorsal stream during SIN perception, we performed the ROI analysis in BOLD activation, TiFC (beta coefficients for the PPI regressors), and RSFC.

- Why an ad hoc non parametric analysis is needed to test the contrast (ONM - YNM <>  OM - YNM)?  Can't this be done in the context of the prametric statistical framework of the main analyses (ANOVA, linear mixed effects modeling)?

**Response:** Thank you for raising this important methodological point. We used a nonparametric bootstrap approach because standard ANOVA or linear mixed-effects models do not directly test whether the difference between ONMs and YNMs is significantly larger (or smaller) than the difference between OMs and YNMs. While these methods can compare individual group means (e.g., ONM vs. OM), they typically do not provide a direct assessment of the relative magnitude of two between-group differences. The bootstrap method allowed us to compute a confidence interval for the difference of differences, that is, (ONM−YNM)−(OM−YNM), over 10,000 iterations. This provided a straightforward, nonparametric way to assess whether the observed difference of differences was distinct from zero. Given our specific hypothesis regarding how ONMs and OMs differ relative to YNMs, this approach was more appropriate than standard parametric tests alone. We have clarified these points in the Methods section to highlight the rationale for using bootstrap analysis.

Line 513-518: Standard parametric tests (e.g., ANOVA or linear mixed models) do not directly assess whether the magnitude of the difference between ONM and YNM exceeds that between OM and YNM. To determine whether the difference between YNMs and ONMs ($\Delta_{ONM-YNM}$) was larger/smaller than the difference between YNMs and OMs ($\Delta_{OM-YNM}$), we compared the two mean differences over left or right ROIs directly and employed a nonparametric bootstrap approach with 10,000 iterations to quantify the uncertainty in this comparison.

- The analysis based on the spatial alignment of the connectivity patterns needs a rationale justification. I am not convinced that some shift in the mean coordinate of a few voxels across groups can be easily interpreted? What would be the neural substrate for this observation?
**Response:** Thank you for the comment. Our coordinate-based analysis revealed the central position of voxels with the highest functional connectivity to the auditory cortex. We found that older non-musicians showed reduced spatial alignment to young adults compared to older musicians. This approach allowed us to directly quantify the spatial reorganization of connectivity patterns with aging. Specifically, older non-musicians showed a superior shift along the Z-axis in the left superior precentral gyrus, suggesting altered spatial organization of peak connectivity.

We have expanded the results section to further justify the coordinate-based analysis.

Line 229-233: Next, we extended our analysis to quantify spatial shifts in connectivity peaks within the left PrCGsup. While ISPC measures overall spatial neural alignment between older and young adults, it does not capture specific reorganization patterns. By extracting the mean coordinates of the top 10% of voxels with the highest TiFC, we precisely mapped connectivity spatial shifts in older adults relative to young non-musicians (**Fig. 4D**).

To our knowledge, few studies, with the exception of Srokova et al [1], have investigated aging-related shifts in peak neural activity, making our interpretation preliminary. We speculate that this superior shift reflects an age-related cortical reorganization, potentially aligning with the “compensation by reorganization” hypothesis [2], that may be driven by age-related alterations in white matter integrity between auditory and motor areas. Importantly, older musicians did not exhibit this shift, suggesting that long-term musical training preserves a more youth-like connectivity topography. However, the absence of behavioral correlations implies that this shift marks age-related functional reorganization rather than effective compensation. We have clarified these points in the discussion and emphasized the need for future structural evidence.
Line 359-370: Furthermore, by quantifying the precise spatial shifts in connectivity peaks using the coordinate-based analysis, we extended our analysis beyond ISPC’s alignment index. While the ISPC measure provided an overall index of spatial alignment, it could not reveal specific reorganization details. Consistent with previous study using a memory task [51], our findings suggest systematic age-related reorganization of functional connectivity, possibly due to alterations in white matter integrity between auditory and motor regions. The absence of this shift in OMs implies that long-term musical training may help maintain a youth-like spatial configuration of neural connectivity. This preserved spatial pattern, together with more youth-like overall connectivity strength, supports the “Hold-back upregulation” hypothesis, wherein cognitive reserve modulates both the magnitude and fine-scale topography of task-induced functional connectivity during SIN perception. However, additional structural evidence is needed to fully understand the neural substrate underlying these spatial shifts.


Reviewer #2: Dear Authors,
thank you for the wonderful work done! Nevertheless, some additional questions and comments:

**Response:** Thank you for positive comments and constructive suggestions on the theoretical framework. Here are point-to-point responses to your suggestions.

1) Introduction should include more detailed explanation why you expect differences between groups based on occupation and age. It is unclear in the current text.

**Response:** Thank you for the suggestion. We have revised the Introduction to more clearly explain our expectations regarding group differences based on occupation and age.

Line 55-58: According to the Posterior-Anterior Shift in Aging and Compensation-Related Utilization of Neural Circuits model [8,9], **older adults would exhibit upregulated neural activity in frontoparietal brain regions more extensively than younger adults during cognitive tasks** [10] (**Fig. 1A**).

Line 60-65: The Scaffolding Theory of Aging and Cognition model suggests that positive lifestyle choices, such as musical training, higher levels of education, and bilingualism, contribute to cognitive and brain reserve, which represents the accumulation of cognitive and neural resources before the onset of age-related brain changes [1,6]. **Thus, we hypothesized that individuals with greater cognitive reserve would demonstrate enhanced neural resources than those without such reserve** (**Fig. 1B**).

Furthermore, we have reorganized the Introduction by moving the fourth paragraph before the hypothesis paragraph. This paragraph explains the expected group differences based on the cognitive reserve conferred by musical training and the age-related alternation observed in speech-in-noise perception tasks.

Line 80-86: Prior research revealed enhanced sensorimotor integration, better speech signal encoding and more robust functional connectivity in the auditory dorsal brain region during speech processing [27–31]. **These findings suggest that musicians possess greater neural resources than non-musicians, contributing to their enhanced task performance** (**Fig. 1B)**. In comparison, older adults tend to over-recruit dorsal stream brain regions to compensate for deficits in speech-in-noise (SIN) perception (**Fig. 1A)** [32–34].

These revisions, along with the new arrangement, help clarify our rationale for expecting distinct neural patterns based on age and occupational experience.

2) I would argue that, while occupation (in this case - musician) is an element of cognitive reserve, it can not be used as the only measure of CR. Even more, within the introduction, authors introduce different models of cognitive compensatory ageing, but omit The Cogntive Reserve theory, providing only references to aged publications by Stern et al. If authors are adamant in keeping occupation-musician as the only measure of cognitive reserve, I would definately suggest integrating more information on the actual theory. In addition, current consensus on cognitive reserve is that it should be (a) measured longitudinally and (b) considered as a moderator between the brain and cognitive functioning in case of pathology. Please, refer to the publication by Reserve and Resilience Framework Group: Stern, Y., Albert, M., Barnes, C. A., Cabeza, R., Pascual-Leone, A., & Rapp, P. R. (2023). A framework for concepts of reserve and resilience in aging. Neurobiology of aging, 124, 100-103.

**Response:** Thank you for highlighting this important point. We agree that musical expertise is one type of activity promoting cognitive reserve. Accordingly, we have revised the title and abstract to clarify that the cognitive reserve examined in this study specifically derives from long-term musical training.

Line 41-44: Our findings show that cognitive reserve accrue through long-term music training holds back age-related neural recruitment during speech-in-noise perception and enlighten the intricate interplay between cognitive reserve and age-related upregulated activity during cognitive tasks.

In addition, cognitive reserve may help compensate for life-course related brain changes. This theoretical framework aligns well with our hypotheses. In our revised Introduction, we have expanded the discussion to integrate cognitive reserve theory.

Line 94-98: Cognitive Reserve theory suggests that experiences, especially in childhood and throughout life, contribute to building a “reverse” of cognitive abilities, which can help mitigate the impact of age-related brain decline [38]. We propose two hypotheses regarding how accumulated reserve from long-term musical training interacts with upregulated neural activity when older adults perform SIN perception tasks.

We appreciate the reviewer’s suggestion and believe these revisions better situate our work within the current framework of cognitive reserve research, including insights from Stern et al. (2023).

3) Comments and recommendations regarding participants:
- did you also consider specific areas of music? There are studies suggesting instrument-specific activities.

**Response:** In our previous behavioral study, we compared the effects of long-term vocal and instrumental training on speech-in-noise perception in both young and older adults and did not find significant differences between these groups [3]. Based on these findings and older musicians who were eligible and willing to participate in our fMRI study are rare, we did not recruit musicians based on specific musical training types. However, we agree that instrument-specific neural activities could provide valuable insights, and we acknowledge this as a limitation that should be addressed in future research.

Line 399-404: All OMs were pooled regardless of training type (e.g., piano, voice, violin). Different training modalities engage distinct sensorimotor networks. Singing primarily engages articulatory control, while piano performance recruits fine finger motor circuits. Future studies comparing older adults with type‑specific expertise could clarify how different forms of musical training contribute to cognitive reserve in SIN perception.

- considering the size of the sample, replacing outliers with the group average might not be the best approach, as it increases the risk of Type I error (e.g. see Gress, T. W., Denvir, J., & Shapiro, J. I. (2018). Effect of removing outliers on statistical inference: implications to interpretation of experimental data in medical research. Marshall journal of medicine, 4(2), 9. and Vankov, I. I. (2023). The hazards of dealing with response time outliers. Frontiers in Psychology, 14, 1220281. for differences in outlier treatment methods). While I do not claim this is the case, my suggestion would be to triangulate the data analysis using different approaches to removing outliers, to ensure that the correct approach has been chosen.

**Response:** Thank you for raising this important point. To address this concern, we have now triangulated our data analysis using alternative outlier treatment methods. In line with Vankov’s recommendations [4], we employed two common outlier treatment methods, replacing outliers with the group median and truncating outliers. We did not apply logarithmic or inverse transformations to our data because our data are not suitable for these methods (contain negative values). These additional analyses confirmed that our main findings remain robust regardless of the outlier handling method used.

**Response Table 1**. Mixed-design ANOVA and post hoc analysis of task-induced functional connectivity. Outliers are **replaced with the group median**.

| Seed: LSTG | Target regions | Group main effect  $F_{2,69}$($P_{fdr}$) | OMs vs. ONMs  $t_{69}$($P_{fdr}$) | OMs vs. YNMs  $t_{69}$($P_{fdr}$) | ONMs vs. YNMs  $t_{69}$($P_{fdr}$) |
| --- | --- | --- | --- | --- | --- |
|  | L SMA | 16.00(<0.001) | 2.05(0.044) | -3.54(0.001) | -5.59(<0.001) |
|  | L SMG | 12.64(<0.001) | 2.38(0.020) | -2.64(0.015) | -5.03(<0.001) |
|  | L PrCGsup | 25.02(<0.001) | 2.46(0.016) | -4.51(<0.001) | -6.97(<0.001) |
|  | L SM | 13.55(<0.001) | 0.63(0.533) | -4.16(<0.001) | -4.79(<0.001) |
|  |  |  |  |  |  |
| Seed: RSTG | Target regions |  |  |  |  |
|  | R SMA | 7.10(0.002) | 2.24(0.042) | -1.50(0.137) | -3.74(0.001) |
|  | R SMG | 18.83(<0.001) | 2.68(0.009) | -3.44(0.001) | -6.12(<0.001) |
|  | R PrCGsup | 15.39(<0.001) | 3.13(0.004) | -2.40(0.019) | -5.53(<0.001) |
|  | R SM | 3.69(0.030) | 1.85(0.104) | -0.80(0.425) | -2.65(0.030) |

**Response Table 2**. Mixed-design ANOVA and post hoc analysis of task-induced functional connectivity. Outliers are **truncated**.

| Seed: LSTG | Target regions | Group main effect  $F_{2,69}$($P_{fdr}$) | OMs vs. ONMs  $t_{69}$($P_{fdr}$) | OMs vs. YNMs  $t_{69}$($P_{fdr}$) | ONMs vs. YNMs  $t_{69}$($P_{fdr}$) |
| --- | --- | --- | --- | --- | --- |
|  | L SMA | 9.63(<0.001) | 1.34(0.183) | -2.95(0.007) | -4.29(<0.001) |
|  | L SMG | 9.52(<0.001) | 1.90(0.062) | -2.45(0.025) | -4.35(<0.001) |
|  | L PrCGsup | 15.32(<0.001) | 2.02(0.048) | -3.46(0.001) | -5.47(<0.001) |
|  | L SM | 8.47(0.001) | 0.02(0.980) | -3.55(0.001) | -3.58(0.001) |
|  |  |  |  |  |  |
| Seed: RSTG | Target regions |  |  |  |  |
|  | R SMA | 6.60(0.003) | 2.12(0.057) | -1.50(0.139) | -3.62(0.002) |
|  | R SMG | 13.25(<0.001) | 2.20(0.031) | -2.93(0.007) | -5.13(<0.001) |
|  | R PrCGsup | 8.73(0.001) | 2.44(0.026) | -1.72(0.090) | -4.16(<0.001) |
|  | R SM | 3.08(0.052) | 1.74(0.130) | -0.67(0.507) | -2.41(0.057) |

The following analyses including testing whether the difference between ONMs and YNMs exceeds that between OMs and YNMs, correlation analysis, spatial alignment analysis, resting-state functional connectivity analysis, and BOLD activation analysis. None of those analyses was affected, as no outliers (defined as values exceeding 2 standard deviations from the mean) were detected.

Since the median is a measure of central tendency which is less sensitive to the presence of extreme values, Vankov [4] suggests using the median to replace outliers instead of the mean. We followed this recommendation and replaced all outliers with the group median in the revised analysis.

4) Regarding data analysis:
- while exclusion of education as is discussed in the supplementary text, it should be discussed more in the body of the text as well. It should also be clarifies, what is meant by "the self-reported years of education cannot represent the education level of the older subjects reliably". It might not represent the level of knowledge, however, it can quantify the formal and informal education, which is the most common way of measuring education (even if not the best). In any case, I would suggest to briefly clarify this in the body of the text as well.

**Response:** Thank you for the comments. While self-reported years of education are commonly used to quantify formal and informal education, our interviews with older musicians revealed fundamental qualitative differences in their educational backgrounds compared to older non-musicians. Many musicians attended specialized institutions, such as music middle schools, music high schools, or conservatories, where the curricula primarily focus on music training rather than the broader academic subjects (e.g., language and science) emphasized in standard education. Therefore, even when the duration of education was comparable between groups, the educational experiences were not equivalent. We have expanded the explanation in the Supplementary Materials and included a brief clarification in the Results section.

Results section Line 144-147: “While we observed differences in years of education between older musicians and non-musicians, supplementary analyses of covariance to assess the effect of education confirmed that years of education did not significantly influence our behavioral or neural measures, ruling it out as a confounding factor (for details, see Supplementary Text).”

Supplementary Materials line 17-23: “Importantly, our interviews revealed fundamental qualitative differences in educational backgrounds between older groups. Most older musicians attended specialized institutions, such as music middle schools, music high schools, or conservatories, with curricula focused on music training rather than on the broader academic subjects (e.g., language and science) typical of standard education. This qualitative difference means that years of education cannot be directly compared between musicians and non-musicians, even when the nominal duration is similar. Consequently, self-reported years of education do not reliably represent educational attainment in our older cohort.”

- In general, Results section is well structured and appropriate data analysis methods are used.

**Response:** Thanks.

5) Discussion:
- while the authors have presented their results within the context of the current literature, the limitations of the study are not really discussed. I would encourage the authors to address the impact of the issues like sample size and characteristics, research design, task type etc. on the study results.

**Response:** Thank you for the suggestions. We have now incorporated a discussion of several limitations in the Discussion section.

Line 394-407: Our study has some limitations. First, we cannot establish cause-and-effect relationships between cognitive reserve from musical expertise and age-related changes in SIN perception using a cross-sectional design. Second, the investigation focused solely on the cognitive reserve derived from musical training and its effect on one specific task, SIN perception. It is unclear whether our findings generalize to other sources of cognitive reserve and other cognitive functions. Moreover, our sample size was relatively modest, limiting statistical power. All OMs were pooled regardless of training type (e.g., piano, voice, violin). Different training modalities engage distinct sensorimotor networks. Singing primarily engages articulatory control, while piano performance recruits fine finger motor circuits. Future studies comparing older adults with type‑specific expertise could clarify how different forms of musical training contribute to cognitive reserve in SIN perception. Future studies should aim to use larger, more diverse samples, longitudinal designs, and alternative task paradigms to further elucidate the interplay between cognitive reserve and age-related neural changes. Future studies should explore how varying task difficulty (e.g., different SNRs) interacts with cognitive reserve to shape age‑related changes in neural activity.

- to add to the comment at the beginning, the concept of cognitive reserve in the discussion section is used very loosely.

**Response:** Thank you for the comments. We have expanded the discussion combining our result with the concept of cognitive reserve raised in cognitive reserve theory.

Line 332-347: Cognitive Reserve theory posits that “reserve” accrued through experience and training can help cope with or compensate for life-course related brain changes, leading to better-than-expected cognitive performance [1,2,38]. Our results are consistent with this framework, as cognitive reserve derived from musical training helps older musicians maintain superior SIN perception performance relative to older non-musicians. Furthermore, our study expands cognitive reserve theory by providing evidence of a potential neural mechanism underlying its protective effects. Specifically, cognitive reserve derived from long-term musical training appears to help preserve a youth-like neural activity pattern in key auditory and sensorimotor regions, which is associated with better-than-expected SIN perception performance in older adults. This finding suggests that, beyond merely compensating for age-related declines, cognitive reserve may work by maintaining the integrity and functional architecture of neural networks, thereby mitigating the adverse effects of aging on cognitive performance. Future studies should further test this “Hold-back upregulation” hypothesis using different cognitive tasks, such as memory and attention tasks, and investigate other sources of reserve, such as physical exercise and bilingualism. Additionally, examining how different types of learning transfer may influence such interactions will be important for generalizing the findings.

Overall, this is a well-structured article with well conducted data analysis, however, some work on the theoretical framework is suggested.

**Reference**

1.	Srokova S, Hill PF, Rugg MD. The Retrieval-Related Anterior Shift Is Moderated by Age and Correlates with Memory Performance. J Neurosci. 2022;42: 1765–1776. doi:10.1523/JNEUROSCI.1763-21.2021

2.	Cabeza R, Albert M, Belleville S, Craik FIM, Duarte A, Grady CL, et al. Maintenance, reserve and compensation: the cognitive neuroscience of healthy ageing. Nat Rev Neurosci. 2018;19: 701–710. doi:10.1038/s41583-018-0068-2

3.	Zhang L, Fu X, Luo D, Xing L, Du Y. Musical Experience Offsets Age-Related Decline in Understanding Speech-in-Noise: Type of Training Does Not Matter, Working Memory Is the Key. Ear and Hearing. 2021;42: 258–270. doi:10.1097/AUD.0000000000000921

4.	Vankov II. The hazards of dealing with response time outliers. Front Psychol. 2023;14. doi:10.3389/fpsyg.2023.1220281
